# Optimalization of preparation of apo-cytochrome b_5_ utilizing apo-myoglobin

**DOI:** 10.2478/v10102-010-0037-8

**Published:** 2010-11

**Authors:** Barbora Mrázová, Markéta Martínková, Václav Martínek, Eva Frei, Marie Stiborová

**Affiliations:** 1 Department of Biochemistry, Faculty of Science, Charles University, Prague, Albertov 2030, 128 40 Prague 2, CZECH REPUBLIC; 2 Division of Molecular Toxicology, German Cancer Research Center, In Neuenheimer Feld 280, 69120 Heidelberg, GERMANY

**Keywords:** apo-myoglobin, cytochrome b_5_, butanon extraction, pH

## Abstract

Cytochrome b_5_ (cyt b_5_), a component of endoplasmic reticulum membrane, plays a role in modulation of enzymatic activity of some cytochrome P450 (CYP) enzymes. The effect of apo-cytochrome b_5_ on this enzymatic system has not been investigated in details, because preparation of cyt b_5_ as a pure protein failed in many laboratories. In order to prepare the native apo-cytochrome b_5_ in a large scale we utilized a protein with higher affinity toward the heme; the apo-myoglobin from the equine skeletal muscle. In the first step, we extracted heme moiety from the native myoglobin by butanone extraction. Than the effect of pH on spontaneous heme release from both proteins was investigated: purified rabbit cyt b_5_ as well as equine skeletal muscle myoglobin. The prepared apo-myoglobin was incubated with the cyt b_5_ and heme transfer was monitored as a shift of absorption maximum from 413 to 409 nm in pH varying between 3–6 (10 mM KH_2_PO_4_, pH 3–6). Here, we obtained 43 mg of the equine skeletal muscle apo-myoglobin (43% yield). The optimal pH range for heme transfer from cyt b_5_ into apo-myoglobin was between 4.2 and 5. Native apo-cytochrome b_5_ was successfully prepared using procedure described here.

## Introduction

Cytochrome b_5_ (cyt b_5_), a component of endoplasmic reticulum membrane, is a heme protein with molecular weight of 16,800. It is composed of two functional domains, a soluble heme-containing core, and a short hydrophobic C-terminal tail, which anchors the protein into the microsomal membrane (Schenkman and Jansson, 2003). Cyt b_5_ has been shown to stimulate, inhibit or have no effect on cytochrome P450 (CYP)-mediated reactions. There are two major theories explaining this effect: (i) direct electron transfer from cyt b_5_ to CYP and (ii) conformational effects of cyt b_5_ on CYP without contribution of electron transfer (Yamazaki *et al*., 2001). Recently, we found that cyt b_5_ isolated from rabbit liver microsomes and reconstituted with CYP1A1 and NADPH:CYP reductase modulates the oxidation of carcinogenic azo dye Sudan I by this system (Stiborova *et al*., 2006).

To study the mechanism of this modulation, not only the effect of native cyt b_5_, but also that of the apo-cytochrome b_5_ (lacking the electron transferring cofactor – heme) on Sudan I oxidation is necessary to be evaluated.

The affinities of apohemoproteins for heme are very large, showing equilibrium dissociation constants in the 10^–10^–10^–15^ M region (Hargrove *et al*., 1996; Miksanova *et al*., 2006). In the holoproteins, the heme prosthetic group appears to be stabilized by a large number of hydrophobic (van der Waals) and electrostatic contacts. The vinyl groups are pointing toward the protein interior and surrounded by nonpolar aliphatic and aromatic side chains, whereas the propionates point toward the solvent and interact with a variety of charged or polar amino acids. These interactions and the extremely small dissociation constants imply a high degree of specificity in the binding process (Hargrove *et al*., 1994). However, a variety of experimental evidence suggests that the association of heme with apoglobin is little affected by globin structure (Hargrove *et al*., 1996).

In the present time there are just two methods for the apohemoproteins preparation in a large scale. (i) First it is a butanone extraction method (Rossi-Fanelli *et al*., 1959). (ii) Second it is a expression of recombinat hemoprotein in the system without heme precursor – δ-aminolevulic acid (Miksanova *et al*., 2006). However, the first method requires nonphysiological conditions such as pH 2.5 and the second method cannot guaranty that whole amount of the expressed hemoprotein would be present in its apoform. The *E. coli* used as an expression system contains its own heme, which can be incorporated into the produced hemoprotein. Therefore, here we present the new method for apohemoproteins preparation. Most important are the physiological conditions of the new method and the negligible amount of the residual holohemoprotein.

## Materials and methods

### Preparation of the apo-myoglobin from the equine skeletal muscle

In the first step, heme moiety was extracted from the native myoglobin by butanone extraction (Rossi-Fanelli *et al*., 1958). pH of the myoglobin solution (100 mg of the equine skeletal muscle myoglobin in 40 ml dist. H_2_O) adjusted to pH 2.5 by 1 M HCl. The solution was moved to the separation funnel and equal volume of 2-butanone was added. Mixture was slowly shaked 3 times for 5 minutes and than placed for 10 minutes into the cold room (8°C). Aqueous phase was dialyzed at 8°C against 2 l of water, than 2 days against 2 l of 10 mM Tris, pH 8.0. The solution was concentrated in an Amicon stirred cell using a PM-10 membrane. In order to prevent the precipitation of the apo-myoglobin, pH was adjusted to 5 using 2 M CH_3_COOH before it was stored at –80°C.

### Protein analysis

Protein concentration was determined by the standard bicinchoninic acid protein assay (Wiechelman *et al*., 1988). Bovine serum albumin was used as a standard.

### Spectrophotometric measurements

Spectrophotometric measurements were performed on a Hewlett Packard 8453 UV spectrophotometer with 1 ml samples. 10 µl of purified rabbit cyt b_5_ or equine skeletal muscle myoglobin was diluted to 990 µl of 10 mM KH_2_PO_4_, pH 3–5. Afterwards the cyte b_5_ was incubated with the apo-myoglobin in the molar ratio 1:1.5. The heme transfer in various pH was monitored as a shift of absorption maximum of heme from 413 to 409 nm. The exact pH values of individual incubation mixtures were measured after the spectrophotometric measurement.

## Results

**Figure 1:**
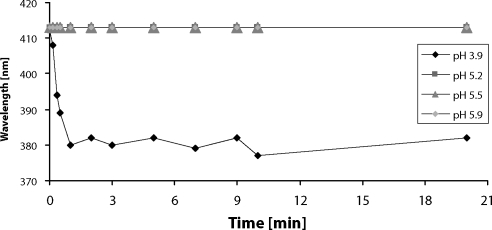
The effect of pH on spontaneous heme release from purified rabbit cytochrome b_5_.

The effect of pH on spontaneous heme release from rabbit cyt b_5_ and equine skeletal muscle myoglobin was investigated. Figure 1 shows the low pH induced dissociation of heme from cyt b_5_. The absorption maximum of heme shifts, during incubation at pH 3.9, from 413 nm to 382 nm indicating release of the free heme cofactor. This process was fast, cyt b_5_ lost its heme completely during the first minute of incubation. On the contrary, the heme bound in molecule of cyt b_5_ is stable at pH between 5.2–5.9 (absorption maximum stays at 413 nm).

**Figure 2:**
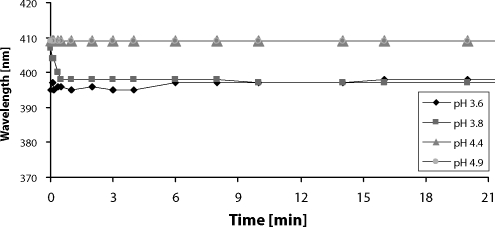
The effect of pH on heme dissociation of equine skeletal muscle myoglobin.

Under the more acidic conditions (pH 3.6–3.8) heme cofactor readily dissociated from the molecule of myoglobin. This process was observed as a shift of heme absorption maximum from 409 to 397 nm within 30 s of the incubation (Figure 2). No changes in absorption maximum of myoglobin were detected for pH 4.4–4.9 (Figure 2).

**Figure 3:**
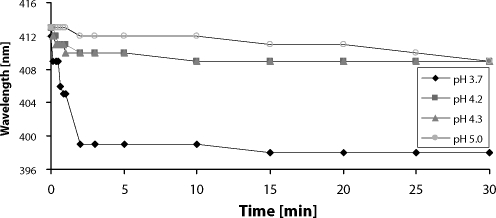
The effect of pH on heme transfer or release from cytochrome b_5_ to apo-myoglobin. Cytochrome b_5_ (2.1 mg/ml) was mixed with apo-myoglobin (3.6 mg/ml) to molar ratio 1:1.5 in phosphate buffer (10 mM KH_2_PO_4_) pH 3-6.

Significant changes in UV/VIS spectra were observed during incubation of cyt b_5_ with apo-myoglobin in various pH. The initial spectrum eliciting maximum at 413 nm corresponds to pure cyt b_5_. Both proteins are loosing heme spontaneously at pH 3.7, therefore the heme transfer at this pH was not observed (Figure 3), and the resulting spectrum resembles spectrum of the free hemin.

**Figure 4:**
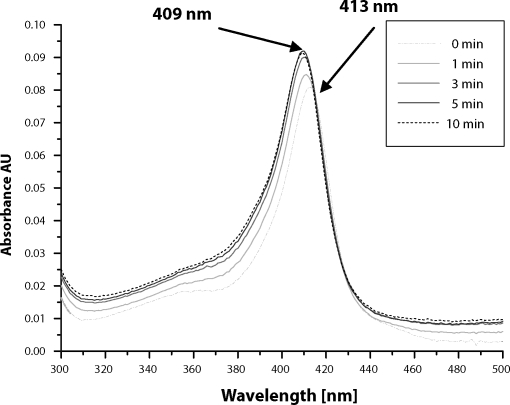
Spectral changes observed during heme transfer from cytochrome b_5_ to apo-myoglobin at pH 4.2.

**Figure 5:**
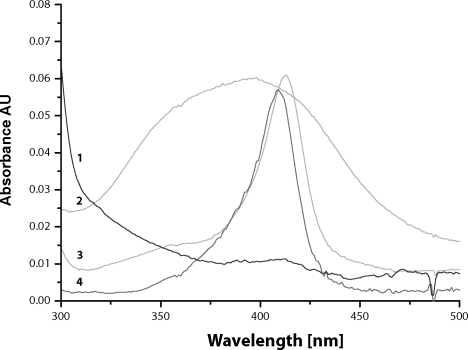
UV/VIS spectra of individual species. (**1**) spectrum of the equine skeletal muscle apo-myoglobin, (**2**) spectrum of hemin with maximum absorbance at ~385 nm, (**3**) spectrum of purified rabbit cytochrome b_5_ with maximum absorbance at 413 nm, (**4**) spectrum of the equine skeletal muscle myoglobin with maximum absorbance at 409 nm.

The absorption maximum of heme chromophore in the incubation mixtures (pH 4.2, 4.3 and 5) was shifted from 413 nm to 409 nm (Figure 3). This finding indicates that heme transfer from cyt b_5_ to apo-myoglobin occurs. Detail spectral changes, observed in incubation mixture pH 4.2, are shown in Figure 4. The resulting spectrum resembles the holo-myoglobin spectrum (maximum 409 nm) (Figure 5), this is indicating that equilibrium state was achieved. Although, there was only slight excess of myoglobin over cyt b_5_ (molar ratio 1:1.5), the heme transfer should be nearly complete ~99%, due to a significantly higher affinity of myoglobin to heme cofactor, ~2 orders of magnitude more than cyt b_5_.

The heme transfer from cyt b_5_ to apo-myoglobin is much slower compared to release of free hemin observed in more acidic conditions (pH 3.9 and 3.8, for cyt b_5_ and myoglobin, respectively). This heme transfer is also extremely sensitive to pH. It tooks 10 minutes at pH 4.2 and 4.3, but at slightly less acidic conditions (pH 5), the transfer was completed in 30 min (Figure 3). Besides, heme transfer from cyt b_5_ to apo-myoglobin at pH 5.25 is extremely slow and required 6 hours to complete (data not shown).

## Discussion

In this study we utilized the principle of the assay for hemin dissociation rate constant (Hargrove *et al*., 1994, 1996) for the preparation of the apohemoprotein in a large scale. The method employs the fact that apomyoglobin has a very high affinity for heme when compared to others hemoproteins (Hargrove *et al*., 1996; Miksanova *et al*., 2006). For example, heme dissociation rate constant for myoglobin is 8.4 × 10^–7^/s (Hargrove *et al*., 1996), however for the bovine cyt b_5_, which is highly homologous (83.5% identity) to rabbit cyt b_5_ used in this study, is 7.7 × 10^–5^/s (Altuve *et al*., 2004). Considering the fact that association of heme with apoglobin is little affected by globin structure (Hargrove *et al*., 1996), the heme equilibrium dissociation constant for myoglobin is at least two orders of magnitude smaller when compared to cyt b_5_, showing much higher affinity to heme.

In order to prepare the apo-cytochrome b_5_ in a large scale we utilized a protein with high affinity toward the heme; the apo-myoglobin from the equine skeletal muscle. We obtained 43 mg of the equine skeletal muscle apo-myoglobin (43% yield). We investigated the effect of pH on spontaneous heme release from both proteins: purified rabbit cyt b_5_ as well as equine skeletal muscle myoglobin. These realize shows that the optimal pH for heme transfer from cyt b_5_ into apo-myoglobin is 4.2–5.0.
